# Drug-Loaded Lipid Magnetic Nanoparticles for Combined Local Hyperthermia and Chemotherapy against Glioblastoma Multiforme

**DOI:** 10.1021/acsnano.3c06085

**Published:** 2023-09-12

**Authors:** Lilianne Beola, Nerea Iturrioz-Rodríguez, Carlotta Pucci, Rosalia Bertorelli, Gianni Ciofani

**Affiliations:** † Smart Bio-Interfaces, Istituto Italiano di Tecnologia, Viale Rinaldo Piaggio 34, Pontedera 56025, Italy; ‡ Translational Pharmacology, Istituto Italiano di Tecnologia, Via Morego 30, Genova 16163, Italy

**Keywords:** glioblastoma multiforme, lipid magnetic nanovectors, chemotherapy, hyperthermia, multimodal therapy, intratumoral administration

## Abstract

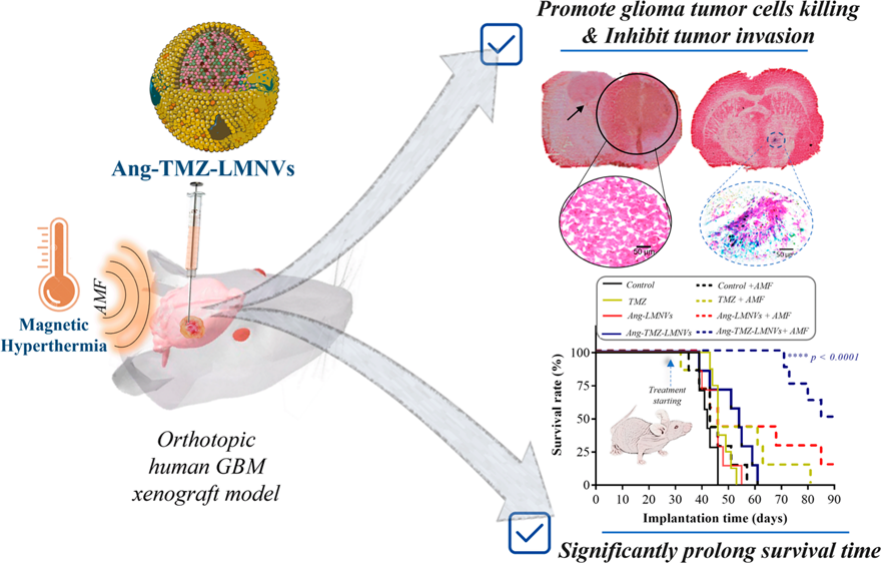

Glioblastoma multiforme (GBM) is a devastating tumor of the central nervous system, currently missing an effective treatment. The therapeutic gold standard consists of surgical resection followed by chemotherapy (usually with temozolomide, TMZ) and/or radiotherapy. TMZ does not, however, provide significant survival benefit after completion of treatment because of development of chemoresistance and of heavy side effects of systemic administration. Improvement of conventional treatments and complementary therapies are urgently needed to increase patient survival and quality of life. Stimuli-responsive lipid-based drug delivery systems offer promising prospects to overcome the limitations of the current treatments. In this work, multifunctional lipid-based magnetic nanovectors functionalized with the peptide angiopep-2 and loaded with TMZ (Ang-TMZ-LMNVs) were tested to enhance specific GBM therapy on an *in vivo* model. Exposure to alternating magnetic fields (AMFs) enabled magnetic hyperthermia to be performed, that works in synergy with the chemotherapeutic agent. Studies on orthotopic human U-87 MG-Luc2 tumors in nude mice have shown that Ang-TMZ-LMNVs can accumulate and remain in the tumor after local administration without crossing over into healthy tissue, effectively suppressing tumor invasion and proliferation and significantly prolonging the median survival time when combined with the AMF stimulation. This powerful synergistic approach has proven to be a robust and versatile nanoplatform for an effective GBM treatment.

## Introduction

Glioblastoma multiforme (GBM) is the most common and deadly brain tumor.^1^ According to the World Health Organization (WHO), primary GBM begins with grade IV (highly aggressive) with no evidence of lower grades. GBM accounts for approximately 79% of all primary central nervous system malignancies and for 2% of all cancers diagnosed annually worldwide.^2^ It is characterized by rapid proliferation, high invasiveness and migration, and devastating neurologic deterioration.^3^ Despite the current aggressive standard therapies, including surgical resection and postoperative radiotherapy (RT) with concomitant and adjuvant chemotherapy (ChT), the median progression-free survival remains extremely low (<7 months).^4^ This dismal outcome is related to the difficulty of completely removing the tumor by surgery and to the resistance of GBM to conventional chemotherapeutic agents due to several factors, including the presence of the blood-brain barrier (BBB)^5^ and the expression of efflux transporters in tumor vessels.^6,7^ In addition, temozolomide (TMZ), the gold standard chemotherapeutic agent for GBM, has a short half-life, so it must be administered in high doses and over a long period of time:^8^ this leads to numerous side effects that significantly affect the quality of life of already debilitated patients. Tumor recurrence or progression is therefore almost inevitable, and no additional survival benefit is observed after completion of the treatment,^9^ resulting in significant morbidity and in a mortality rate of almost 100%—a scenario that has not changed significantly over the past 40 years.

Local tumor treatment is a viable alternative not only for GBM patients who are not candidates for surgical resection, but also to increase the concentration of drugs in the tumor mass and reduce the toxic profile associated with systemic administration.^10−13^ In recent decades, with the advent of nanomedicine, a variety of drug delivery systems have been developed to distribute therapeutic agents locally in diseased area. These nanocarriers, which can be tuned by varying their composition and functionalization, can protect the drug from degradation and allow more precise control of its distribution, providing an effective approach for the treatment of intracranial tumors.^14^ Lipid-based nanocarriers are among the most promising systems for this purpose because of their high biocompatibility, ability to overcome the BBB, improved encapsulation capacity, and the possibility to modify their surface to improve tumor targeting;^15,16^ moreover, these systems can be designed to release their cargo when exposed to a specific stimulus (e.g., pH, enzymes, temperature). As a consequence, various multimodal approaches to antitumor drug delivery have been explored, combining conventional therapeutic strategies (*e.g.,* chemotherapy) with other emerging alternatives such as magnetic hyperthermia (MH), photothermal therapy (PTT), and gene therapy.^17−20^ Among them, MH is a promising strategy to develop a minimally invasive, localized, and remote antitumor treatment.^21−23^ Conversely to conventional pharmacological agents used for cancer treatment or other methods of whole-body or regional hyperthermia, the heating in MH remains highly localized, and it depends on the simultaneous presence of magnetic nanoparticles and their excitation under an external alternating magnetic field (AMF).^24^ This contributes to minimize systemic side effects and allows for a better recovery of the neighboring healthy cells or tissues, since the microenvironment of the treated area is not strongly stressed.^25,26^

In the specific case of brain tumors, MH application has been successfully tested in GBM patients along with RT and ChT to boost the therapy effectiveness.^27,28^ Hence, magnetically responsive lipid nanovectors are some of the most promising systems for controlled drug delivery. To this end, the temperature rise induced by nanoparticle delivery into the tumor has been exploited in a series of *in vitro* studies to develop smart magnetic switches that allow the release of chemotherapeutic agents only in response to a specific stimulus (i.e., AMF).^29−32^ This local heating can in turn be used to promote cell death^33^ and destroy the acellular stroma that structurally supports the components of the tumor niche.^34^

To date, just a few approaches based on magnetically responsive lipid systems have been investigated in preclinical practice.^17,35−37^ Moreover, the existing studies do not fully exploit the synergy between ChT and MH, merged into a single nanoplatform, to improve multimodal local therapies against gliomas. In this work, we focused on a promising multifunctional nanoplatform based on lipid-based magnetic nanovectors (LMNVs) to address the need for a more efficient treatment against GBM. This nanoplatform can target several apoptotic and/or necrotic pathways to overcome different therapeutic resistance mechanisms and to significantly improve the therapeutic outcome. Previous *in vitro* studies by our group using LMNVs^30,31^ modified with the glioma-targeting peptide angiopep-2 (Ang-LMNVs)^32^ have shown that this nanoplatform specifically accumulates in GBM cells and is able to induce apoptosis thanks to the combined effect of magnetic hyperthermia and chemotherapy. To evaluate the therapeutic potential of this multifunctional system *in vivo*, we investigated the efficacy of Ang-LMNVs loaded with TMZ (Ang-TMZ-LMNVs), administered intratumorally in mice with orthotopic human U-87 MG-Luc2 GBM xenografts, on tumor growth inhibition (primary end point) and on overall survival prolongation (secondary end point). Our results showed that the administration of Ang-TMZ-LMNVs combined with MH not only inhibited tumor growth and prolonged survival, yet also displayed long-term intratumoral retention and prevented cancer cell migration. The nanoformulation showed no apparent healthy tissue toxicity, and no adverse effects were observed in association with the administered dose. Cytotoxicity effects due to the synergistic action of ChT, promoted by TMZ loaded in the nanovectors, as well as cell sensitization in response to local heating triggered by exposure to an AMF, were also confirmed by histopathological and flow cytometry analysis.

Concluding, a multimodal therapy with lipid-based magnetic carriers has been effectively tested in an GBM orthotopic model, demonstrating its suitability for glioma treatment after intratumoral administration. Our results suggest that the developed multifunctional nanoplatform has an effective action against GBM *in vivo*, indicating its great potential for a future clinical application in the treatment of this deadly disease.

## Results and Discussion

### Nanovectors for Drug Delivery and Magnetic Hyperthermia

TMZ-loaded magnetic lipid nanovectors functionalized with the peptide angiopep-2 (Ang-TMZ-LMNVs) were tested in a human orthotopic xenograft model in order to evaluate their efficacy against GBM. The synthesis, functionalization, and characterization procedures of these nanovectors, as well as their *in vitro* antitumor and targeting efficiency, have been detailed in our previous works.^30−32^ In brief, to achieve magnetic properties suitable for hyperthermia, 3 nm superparamagnetic iron oxide nanoparticles (SPIONs) and TMZ were encapsulated in a lipid matrix by a previously standardized hot sonication procedure.^30^ The blend of lipids (oleic acid (OA); 1-stearoyl-*rac*-glycerol (GMS); 2-dipalmitoyl-rac-glycero-3-phosphocholine-(DPPC); methoxyl poly(ethylene glycol)-1,2-distearoyl-*sn*-glycero-3-phosphoethanolamine (mPEG-DSPE); *N*-hydroxysuccinimide (NHS)-PEG-DSPE) selected for the biocompatible matrix not only facilitates nanoparticle/drug delivery,^22,30−32,38^ but also provides reactive groups (i.e., the NHS group) that enable functionalization of the nanovectors with angiopep-2 to promote glioma targeting.^32,39,40^ Angiopep-2, derived from the Kunitz domain of aprotinin, exhibits high binding to low-density lipoprotein receptor protein 1 (LRP1), which is expressed in endothelial cells of brain capillaries as well as in glioma cells.^40,41^ In addition, incorporation of the near-infrared fluorescent lipophilic tracer DiR (DiIC_18_(7), 1,1′-dioctadecyl-3,3,3′,3′-tetramethylindotricarbocyanine iodide) into the lipid matrix allows *in vivo* tracking of the nanovectors.

The morphological and magnetic properties of the nanovectors have been characterized in previous studies.^30−32^ TEM images (Figure S1, Supporting Information) confirm the spherical morphology of both Ang-LMNVs and Ang-TMZ-LMNVs (core size of 20 ± 5 nm), as previously found. A negligible coercivity at the working temperature with a saturation magnetization of 25 Am^2^/kg Fe_3_O_4_ has also been previously reported,^30,32^ together with the ability to raise the temperature in pretreated (0.2 mg/mL) U-87 MG cells under AMF stimulation (13 kA/m, 750 kHz), reaching a plateau of 41 °C after 40 min.^31,32^ The stability of Ang-LMNVs and Ang-TMZ-LMNVs under conditions simulating the biological environment was confirmed by dynamic light scattering (DLS) measurements. The hydrodynamic diameter (*R*_d_) and polydispersity index (PdI) were measured over time in Dulbecco’s modified Eagle’s medium (DMEM) with 10% serum (DMEM/fetal bovine serum (FBS); Figure S1, Supporting Information). The results showed that the nanovectors were stable over time and had an average *R*_d_ value of less than 300 nm, with PdI not exceeding 0.38 after one month in biological media (Table S1, Supporting Information). After this time, a decrease in *R*_d_ was evident, likely associated with the onset of a degradation process (Figure S1, Supporting Information). These data are important to consider when selecting the right time frame for AMF stimulation after a single administration of the nanovectors. Previous works have shown that SPIONs can retain their properties for at least one month after administration;^42,43^ however, considering that some morphological features of our systems start to change approximately after 30 days of incubation with culture medium, repeated AMF exposure one month after the single administration would probably not be as efficient as at earlier time points. For this reason, in this work, the onset of AMF stimulation was set at 24 h after local administration.

The hemocompatibility of Ang-LMNVs and Ang-TMZ-LMNVs was also tested by examining their interaction with blood to evaluate their suitability for preclinical and eventual clinical use. Both qualitative and quantitative hemolysis assessment results (Figure S2, Supporting Information) confirmed that no significant hemolytic phenomena occurred after 24 and 72 h of continuous incubation with the nanovectors, indicating their safety at the concentration tested.

These results are consistent with previous studies that have already demonstrated their negligible cytotoxicity in absence of magnetic stimulation on human glioblastoma cells (U-87 MG),^30−32^ human endothelial cells (hCMEC/D3), primary astrocytes (HA), and neuron-like cells (differentiated SH-SY5Y).^31,32^ The ability of Ang-LMNVs to specifically target U-87 MG cells^30^ over brain endothelial cells, astrocytes, and neuron-like cells,^32^ and to efficiently cross an *in vitro* model of the BBB^31,32^ has been also demonstrated under standard static conditions^30^ and by using a fluidic bioreactor based on a multicellular model of the BBB to mimic the brain environment.^31,32^ Studies on the uptake mechanism of Ang-LMNVs showed that after administration endocytosis is the main route of internalization in GBM cells,^32^ where they are partially incorporated into acidic organelles (i.e., lysosomes and late endosomes).^30−32^ Finally, the good loading capacity of the nanovectors (4.1%), with a high release extent after magnetothermal stimulation (61.0% at pH 7.4 and 57.6% at pH 4.5 after 4 h of AMF exposure), was also highlighted.^30^ Concerning *in vitro* therapeutic effects, we already reported enhanced apoptotic (50.0% of apoptotic cells vs 9.4% in the group treated with plain TMZ or 9.3% in the group treated with TMZ-LMNVs without AMF stimulation) and antiproliferative (18% of proliferating cells vs 65% in the group treated with plain TMZ or 66% in the group treated with TMZ-LMNVs without AMF stimulation) phenomena after chronic AMF stimulation (*f* = 742.1 kHz; *H* = 14.58 kA/m).^30−32^

Despite the limited clinical data on the maximum tolerable field amplitude *H* and frequency *f*,^27,28,44−46^ finding the optimal conditions for maximum heating within accepted biological limits is important for the use of magnetically responsive nanovectors *in vivo*. To evaluate the ability of Ang-TMZ-LMNVs aqueous suspension to induce MH within the recommended safety range for *in vivo* application, we investigated the temperature rise in response to different combinations of external AMFs (AMF1: *f* = 468.2 kHz and *H* = 9.7 kA/m, *H* × *f* = 4.5 × 10^9^ A/ms; AMF2: *f* = 334.1 kHz and *H* = 12.7 kA/m, *H* × *f* = 4.2 × 10^9^ A/ms). Measurements of plain Ang-LMNVs were also performed as a control (Figure S3, Supporting Information). The AMF conditions were chosen within the technical limits of our device without exceeding the biological limit proposed in the literature (*H* × *f* < 5 × 10^9^ A/ms).^47,48^ The results showed that Ang-TMZ-LMNVs (10.1 mg/mL) elicited a slight increase (≈1.5 °C) in the temperature of the medium (*T*_t=0_ ≈ 21 °C) after 5 min of stimulation under the conditions of AMF1, reaching a value of ≈39.5 °C after 30 min. In the case of AMF2, a faster increase of ≈6.0 °C was observed after 5 min, and the temperature reached ≈40.5 °C after 30 min of exposure (Figure S3, Supporting Information). Considering the faster temperature rise over time and the rise reached after 30 min (within the 40–43 °C range of mild hyperthermia), as well as the fact that the use of higher frequencies may in turn maximize eddy currents and thus cause nonspecific heating of untargeted tissues,^49^ AMF2 was selected for *in vivo* experiments. It must be taken into account that due to the large number of variables affecting the efficacy of MH on complex biological systems, such as the effective local concentration or the degree of aggregation of the nanovectors after internalization, it is difficult to accurately determine the actual effect of the selected heating power in *in vivo* experiments;^50^ nevertheless, the provided estimation provides at least a hint of the expected final outcome.

### In Vivo Antitumor Effects of Ang-TMZ-LMNVs Promoting Synergistic MH and Chemotherapy

**Figure 1:**
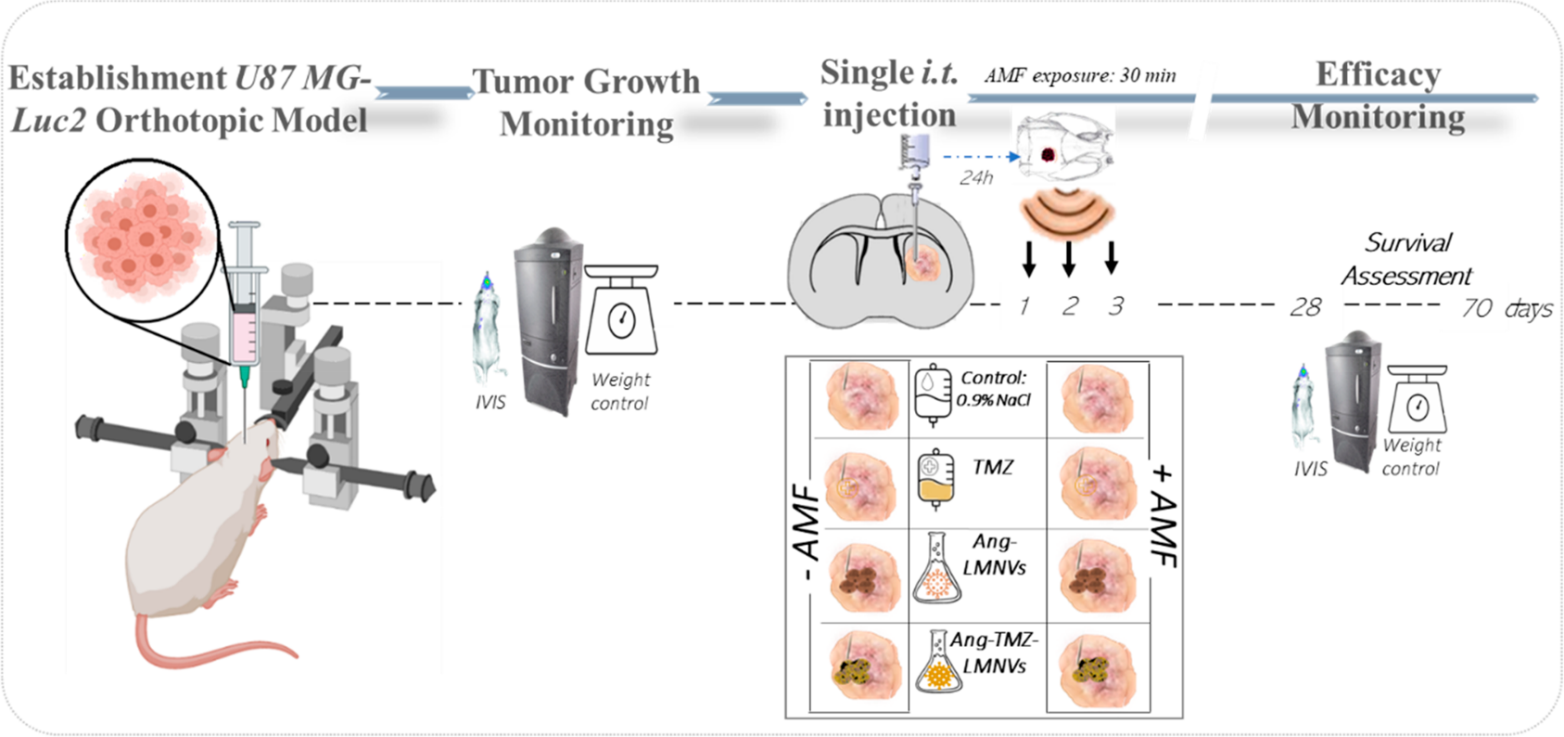
Schematic representation of the experimental procedure, along the different treatment groups and the timeline of the experiments, including AMF exposure and critical end points.

The antitumor effect of our multifunctional nanoplatform was investigated in nude mice bearing orthotopic xenografts of human U-87 MG-Luc2 cells. The selected cell line enables *in vivo* tumor monitoring by bioluminescence and has been shown to form infiltrating and highly proliferative malignant gliomas in immunodeficient mice.^51−53^ Because of the biological differences among individuals, the tumorigenesis rates are different in each mouse. Therefore, the stable disease phase (when mice were randomly selected for treatments) was considered as the time when luminescence levels exceeded 1.5 × 10^7^ p/s/cm^2^/sr (Figure S4, Supporting Information). Mice were treated with a single dose of Ang-TMZ-LMNVs (24 mg/kg_weight_, corresponding to [TMZ] = 0.98 mg/kg_weight_) by intratumoral injection, and then exposed to magnetothermal stimulation (AMF: *f* = 334.1 kHz; *H* = 12.7 kA/m; *t* = 30 min) 24 h after administration and for the following two consecutive days for a total of three exposures. The efficacy of the *in vivo* treatment was evaluated by analyzing the tumor growth and the survival rate of the mice until 70 days after starting the treatment (Figure 1). The body weight of the mice and the general disease symptoms (e.g., focal neurological deficits) were monitored as a control of animal wellness and were thus exploited to define a human end point criterion. Disease progression in mice receiving Ang-LMNVs (24 mg/kg_weight_), free TMZ (0.98 mg/kg_weight_), or saline solution (untreated control, 0.9% NaCl), either exposed or not to AMF, was also monitored to demonstrate the efficacy and the synergy of the proposed treatment (Figure 1).

**Figure 2:**
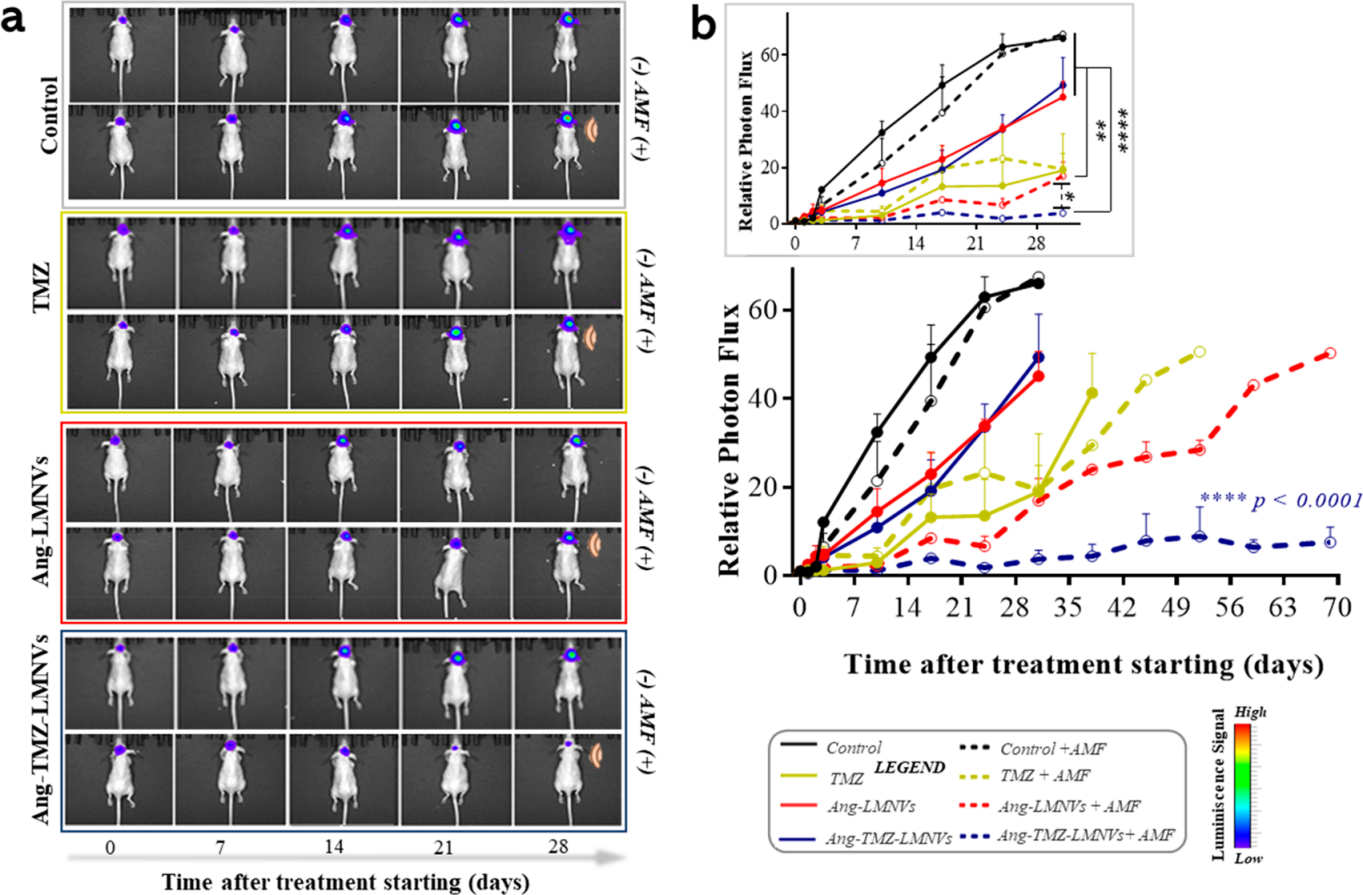
*In vivo* effects of Ang-TMZ-LMNVs promoting MH and drug delivery and tumor growth inhibition. (a) Representative luminescence images of orthotopic U-87 MG-Luc2-based tumors in nude mice after different treatments. (b) Quantitative analysis of luminescence levels at short-term (inlet graph) and long-term after treatments started. Data are expressed as mean ± SD (*n* = 7–8 mice/group; **** *p* < 0.0001, ** *p* < 0.01, * *p* < 0.05, *p* > 0.05 no significance with respect to the control group).

*In vivo* imaging showed that the combined chemo-hyperthermia treatment (Ang-TMZ-LMNVs + AMF) was the most effective one in slowing GBM tumor growth rate, by showing the weakest bioluminescence (average (Avg) radiance ≈ 2 × 10^7^ p/s/cm^2^/sr) among the eight experimental groups (≈15% lower than Ang-LMNVs + AMF, ≈15% lower than TMZ, ≈45% lower than Ang- LMNVs or Ang-TMZ-LMNVs, ≈65% lower than control; Figure 2a). Quantitative analysis was consistent with bioluminescence imaging and confirmed significant inhibition of tumor growth in the mouse brains of the combined treatment group compared to the untreated control (**** *p* < 0.0001) and to individual chemotherapy (TMZ) or MH (Ang-LMNVs + AMF) (* *p* < 0.05; Figure 2b). Although single treatments with TMZ and Ang-LMNVs + AMF significantly slowed tumor growth in the first month after treatment completion (** *p* < 0.01, compared with the untreated control group), significant relapse was observed in the mid- to long-term. This was reflected in a significant increase (≈16%) in the relative photon flux observed in these groups from 28 days after treatment completion; conversely, the group receiving the combined treatment (Ang-TMZ-LMNVs + AMF) showed weak photon signal over time, demonstrating that the therapeutic benefit of the synergistic modality remains stable even after the treatment, in contrast to the individual approaches (Figures 2a,b). Therefore, these strategies alone can induce a cytotoxic effect in tumor cells, as widely described in the literature and in our previous work;^32,54^ nevertheless, just the synergistic approach is able to efficiently inhibit tumor progression. In contrast, the tumors of mice treated with nanovectors without AMF treatment (Ang-LMNVs or Ang-TMZ-LMNVs groups) showed a bioluminescence level similar to that of saline-treated mice (control group) (Figure 2), suggesting no significant effects in terms of tumor growth inhibition. TMZ + AMF and control group + AMF showed similar tumor growth rates with respect to plain TMZ and control groups, confirming that AMF exposure in the absence of magnetically responsive nanovectors has no effect in inhibiting GBM progression, as expected (selected AMF parameters are indeed considered harmless for tissues).^27,28^

**Figure 3:**
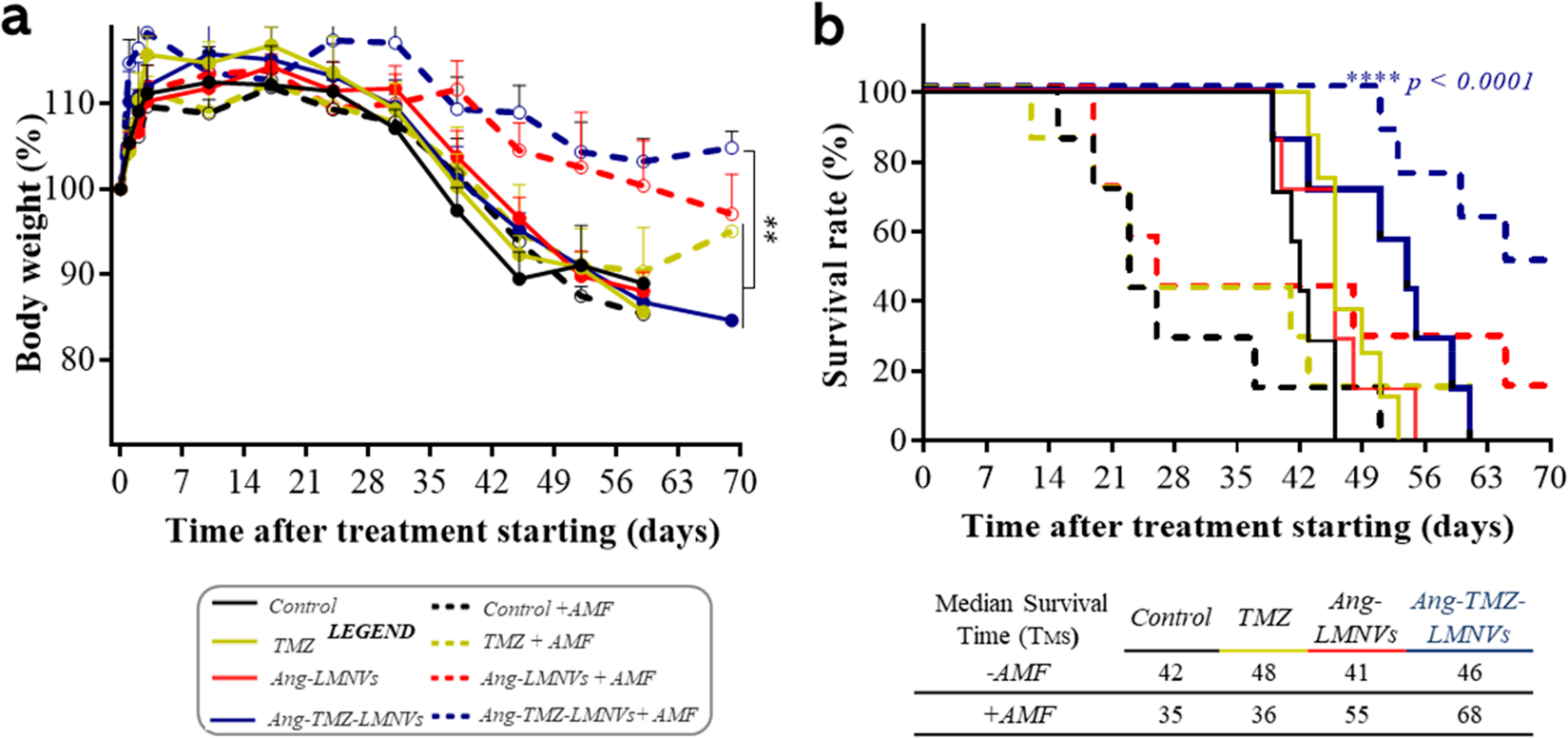
*In vivo* anti-GBM performance of Ang-TMZ-LMNVs promoting MH and delivery of a chemotherapeutic drug. (a) Body weight changes in mice following different treatments. (b) Survival rate of the mice after each treatment. All data are expressed as mean ± SD (*n* = 7–8 mice/group; **** *p* < 0.0001, ** *p* < 0.01, *p* > 0.05 no significance with respect to the control group).

Both the body weight (Figure 3a) and the survival rate (Figure 3b) of tumor-bearing mice were significantly affected by the tumor progression. In mice treated with the synergistic approach (Ang-TMZ-LMNVs + AMF), the body weight barely changed over time, whereas mice treated with hyperthermia and TMZ alone and control animals (saline solution) showed a rapid decrement, possibly due to a quick proliferation and invasion of GBM leading to important overall dysfunctions. Survival curve analysis (Figures 3b) showed that the combined chemo-hyperthermia treatment (Ang-TMZ-LMNVs + AMF) significantly prolonged the median survival time (*T*_MS_) of mice (68 days over the 70 days of total trial observation), and resulted in 50% of the subjects still alive at the end of the experimental protocol compared with control saline-treated group (*T*_MS_ = 42 days, with no animals survived beyond 46 days). Results demonstrate the higher antitumor efficacy of the combined approach with respect to the individual chemotherapy with TMZ (*T*_MS_ = 48 days, with no animals survived beyond 53 days), and to hyperthermia promoted by Ang-LMNVs (*T*_MS_ = 55 days, with only a 14% survival at the end of trial).

*In vitro* experimental evidence shows that the use of magnetically responsive lipid-based nanocarriers may synergistically enhance antiglioma responses,^29−32^ but preclinical evidence are still limited.^16,31−33^ Babincová et al.^17^ showed, in a subcutaneous model of rat glioma based on C6 cells, that a single intratumoral administration of a low-dose free chemotherapeutic agent (doxorubicin) resulted in slower tumor growth, but improved outcomes (sustained tumor regression beyond 28 days after treatment starting) were observed only in the group treated with doxorubicin-loaded magnetoliposomes for integrated chemotherapy and MH. Similary, Aoki et al.^35^ reported the improvement of the therapeutic outcome by combining drug administration (adriamycin) and hyperthermia by using thermosensitive liposomes injected via the tail vein into an intracranial C6 cell-bearing rat glioma model. The main limits of most of the literature studies, including those just mentioned, are however related to the need of repeated nanovector administration and to the challenge to maintain a suitable concentration of nanovectors at the treatment site. Some improvement can be achieved by exploiting passive targeting (i.e., the so-called enhanced permeation and retention effect at tumor site)^36^ and/or with the decoration of the nanoparticle surface with ligands promoting active targeting: as an example, Lu et al.^31^ successfully tested camptosar-loaded magnetoliposomes decorated with cetuximab intravenously administered in an orthotopic U-87 cells tumor model for simultaneous chemotherapy and MH. Nevertheless, achieving durable benefits such as having subjects still alive at the end of the trial protocol is still a challenge, and our findings offer a promising solution in this direction.

### Tumor Colocalization and Nanovector Retention after Local Administration

**Figure 4:**
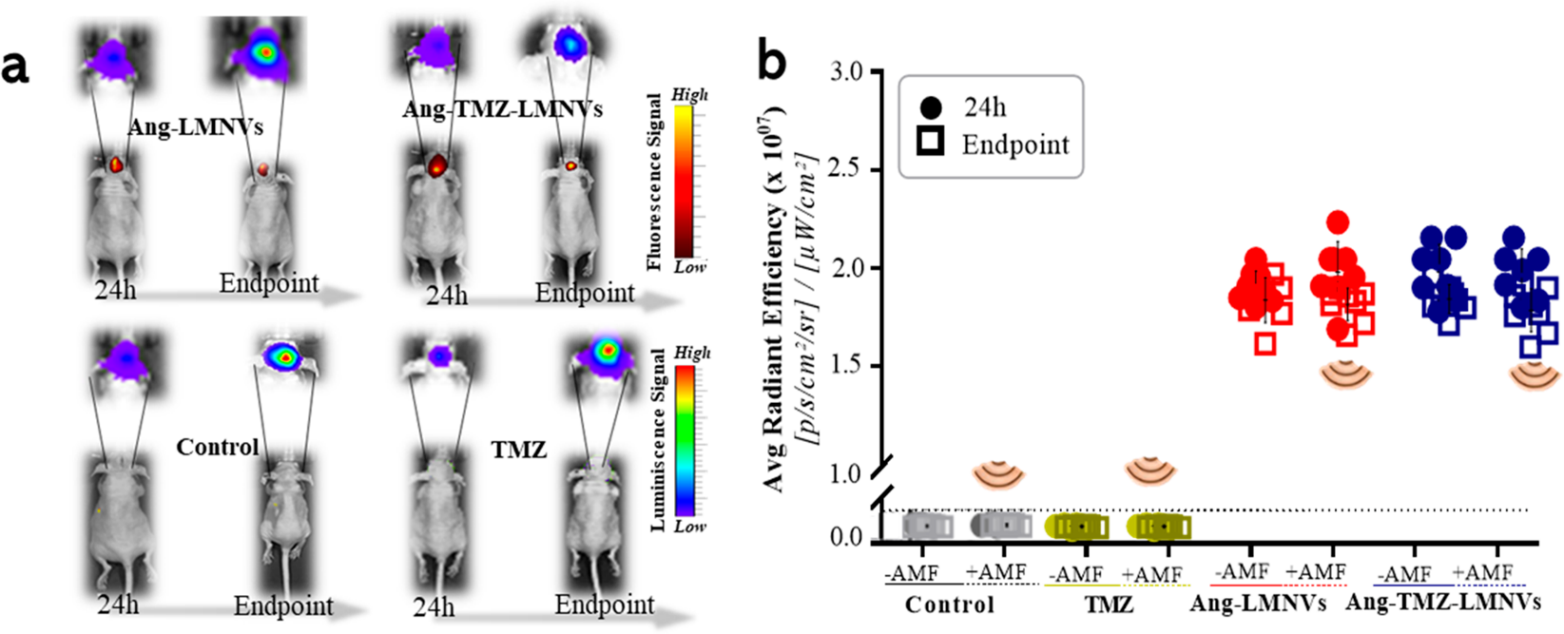
Tumor retention of lipid magnetic nanovectors. (a) *In vivo* epifluorescence and bioluminescence images of representative brains of tumor-bearing mice at 24 h and at the experimental end point after intratumoral injection of 0.9% NaCl saline (control), free TMZ (TMZ), DiR-Ang-LMNVs (Ang-LMNVs), or DiR-Ang-TMZ-LMNVs (Ang-TMZ-LMNVs). (b) *In vivo* DiR signal quantification in tumor-bearing mice at 24 h and at the experimental end point. Results are presented as individual values along with mean ± SD (*n* = 7–8 mice/group).

**Figure 5:**
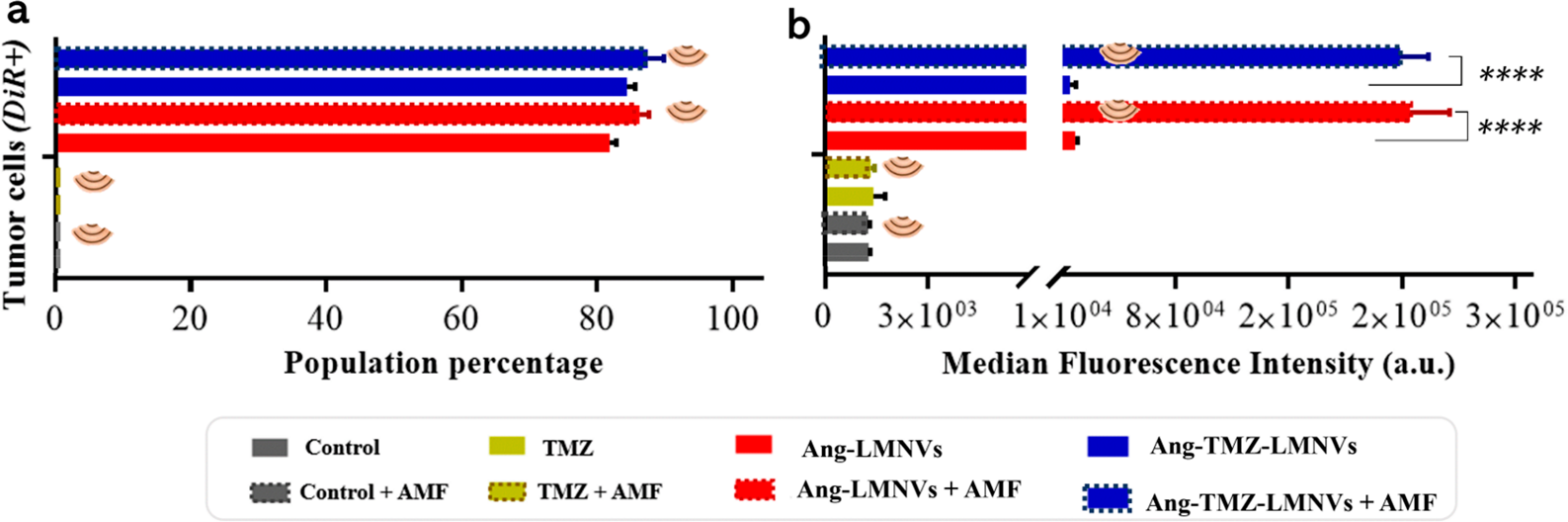
Analysis of cellular uptake efficiency of Ang-LMNVs and Ang-TMZ-LMNVs labeled with DiR measured as (a) percentage of tumor cells containing nanovectors (DiR+) and (b) the changes in the MFI depending on nanovector uptake levels (DiR+) obtained from flow cytometry data. All data are expressed as mean ± SD (*n* = 3 mice/group; **** *p* < 0.0001, *p* > 0.05 no significance).

**Figure 6:**
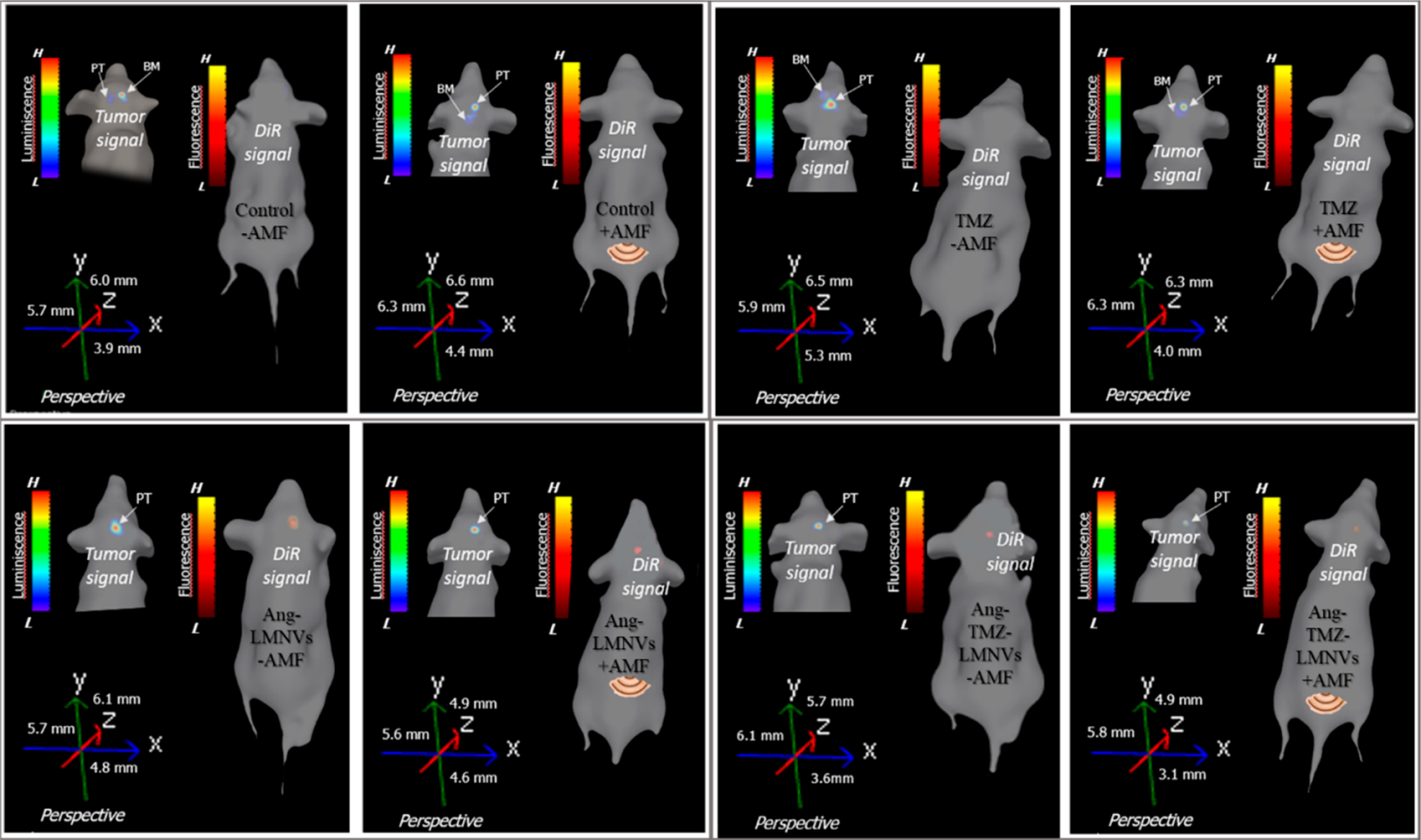
Localization of Ang-TMZ-LMNVs and Ang-LMNVs and estimation of brain tumor size at the experimental end point by *in vivo* 3D fluorescence tomography (FLIT) and 3D diffuse tomography (DLIT), in mice injected with 0.9% NaCl saline (control), free TMZ (TMZ), DiR-Ang-LMNVs (Ang-LMNVs), or DiR-Ang-TMZ-LMNVs (Ang-TMZ-LMNVs) either exposed (+AMF) or not (−AMF) to magnetothermal treatment. Primary tumor: PT; brain metastases: BM.

As we have previously shown, targeted delivery of ChT by local administration of TMZ loaded in magnetic lipid nanovectors within orthotopic tumors represents an effective therapeutic approach in combination with MH. However, it is well-known that one of the major challenges in intratumoral chemo-hyperthermia delivery methods is to achieve homogeneous accumulation of the therapeutic material after injection.^12,13,55−61^ Therapeutic efficacy may be reduced due to the heterogeneity of thermal doses achieved in the tumor once nanovectors are exposed to AMF or due to the presence of areas that do not contain chemoactive agents and escape treatment. In this regard, we further investigated the penetration and tumor retention of our nanovectors after intratumoral administration. To this aim, we used the near-infrared fluorescence dye DiR coupled to Ang-LMNVs and Ang-TMZ-LMNVs for *in vivo* tracking over time (Figure 4) and subsequent *ex vivo* quantification (Figure 5). Nanovectors colocalization with the bioluminescence signal associated with the orthotopic tumor and their retention over time were tracked by two- (Figure 4) and three- (Figure 6) dimensional diffuse tomography (DLIT) and fluorescence imaging tomography (FLIT), respectively, by using an *invivo* imaging system (IVIS). *In vivo* images of the nanovectors were acquired from the beginning of the treatment (24 h after injection) until the end point of the experiment (i.e., when mice were euthanized either because of the development of critical disease symptoms or at the end of the experimental observation period, see Figure 1).

The intracranial injection of the nanovectors was well-tolerated, as indicated by the stable weight of the mice (Figure 3a) and the absence of neurological signs of toxicity after administration. General and focal deficits (*e.g.,* changes in the body symmetry, absence of spontaneous activity, deficiency in the gait and climbing, etc.)^62^ representative of the changes in the mice wellness observed over time were mainly due to the increased tumor burden, since they occurred also in the experimental groups that did not receive the nanovectors. At 24 h after injection of Ang-LMNVs or Ang-TMZ-LMNVs, a strong signal due to DiR fluorescence (Avg radiance_Ang-LMNVs t=24h_ ≈ 1.85 × 10^7^ [p/s/cm^2^/sr]/[μW/cm^2^]*;* Avg radiance_Ang-TMZ-LMNVs t=24h_ ≈ 1.91 × 10^7^ [p/s/cm^2^/sr]/[μW/cm2]), and thus to the presence of the nanovectors, was observed in the brain. These signals remained high throughout all the experimental time frame, until the selected end point (Avg radiance_Ang-LMNVs End point_ ≈ 1.77 × 10^7^ [p/s/cm^2^/sr]/[μW/cm^2^]; Avg radiance_Ang-TMZ-LMNVs End point_ ≈ 1.76 × 10^7^ [p/s/cm^2^/sr]/[μW/cm^2^]) (Figure 4). Moreover, the fluorescence signals of Ang-LMNVs and Ang-TMZ-LMNVs were significantly colocalized with the bioluminescence signal of the orthotopic xenografts during the entire observation period of the experiment (Figure 4a), although some differences were detected over time. At 24 h after administration, the DiR signals of the nanovectors almost completely matched with the bioluminescence area of the tumor cells (Figure 4a), indicating that the delivery process from the injection site to adjacent areas was effective. Conversely, at the final time point, the images showed that the particles were mainly colocalized with the central tumor area, presumably near the injection site (Figure 4a). Considering that our previous stability results suggest that nanovector degradation begins approximately one month after contact with biological media (see Figure S1b and Table S1, Supporting Information), a possible explanation for these data could be associated with the fact that some nanovectors are already degraded at the experimental end point, resulting in a qualitatively stronger signal near the injection site, where they are presumably more concentrated. Also based on our previous *in vitro* studies,^32^ these results suggest that the targeting strategy could play an important role in promoting the specific accumulation of nanovectors in the tumor and in preventing their leakage into healthy tissues. These data may support the hypothesis, encouraged by our previous *in vitro* assays, that the conjugation with the targeting peptide angiopep-2 promotes the retention of LMNVs at the glioma site.^32^

The long-term fate of the nanovectors in each group was also quantified at the end point of the study by flow cytometry. To investigate specific retention in cancer cells, DiR fluorescence signal was evaluated in EpCAM (epithelial cell adhesion molecule)-positive brain tumor cells (Figure S5, Supporting Information), a surface marker that is overexpressed in various neoplasms and is barely detectable in healthy brain cells.^63^ Consistently with the previous findings, these data confirmed a high extent of Ang-LMNV (≈80% positive cells) and Ang-TMZ-LMNV (≈85% positive cells) cellular complexation at the experimental end point (Figure 5a), demonstrating the long-term retention of the nanovectors in the tumor. Notably, despite the percentage of nanovector-positive cells does not significantly change before (Ang-LMNVs and Ang-TMZ-LMNVs groups) and after (Ang-LMNVs + AMF and Ang-TMZ-LMNVs + AMF groups) AMF application, the average amount of nanovectors internalized *per* cell (in terms of median fluorescence intensity -MFI- values) is increased after magnetothermal treatment (MFI_Ang-LMNVs_ ≈ 1.9 × 10^4^ a.u. vs MFI_Ang-LMNVs+AMF_ ≈ 2.5 × 10^5^ au; MFI_Ang-TMZ-LMNVs_ ≈ 1.3 × 10^4^ a.u. vs MFI_Ang-TMZ-LMNVs+AMF_ ≈ 2.1 × 10^5^ au; Figure 5b), suggesting that hyperthermia may promote cellular uptake, even if we cannot exclude effects on drug diffusion through the lipid matrix of the nanoparticles.

Three-dimensional (3D) *in vivo* reconstruction images at the end of the experiment also confirmed the long-term retention of the nanovectors in orthotopic tumors and the enhanced antitumor effect of the combined approach (Figure 6). As we expected, the DiR fluorescence signal of the nanovectors in the FLIT/DLIT reconstructions were strongly colocalized with the bioluminescence of the tumors. 3D tomography reconstruction of the DLIT data sets allowed us to generate bioluminescence images from the transaxial, coronal, and sagittal planes to determine the approximate size of the brain tumor (mm) and to distinguish secondary lesions (metastases). Image analysis revealed significant signals of brain metastases (BM) in the control and free TMZ-treated groups, which were not observed in mice receiving nanovectors (Ang-LMNVs and Ang-TMZ-LMNVs), even in the absence of magnetothermal stimulation (Figure 6). In addition, smaller sizes (millimeters) of the primary tumor (PT) were observed in the combined treatment group (Ang-TMZ-LMNVs + AMF) compared with the saline-treated control groups or with individual ChT (TMZ-treated groups) and MH (Ang-LMNVs + AMF) groups (Figure 6). These data are consistent with the enhanced tumor growth inhibition observed at the end of the study for the combinatorial approach (Figure 2), and confirm the ability of our nanovectors to efficiently treat GBM.

Several preclinical tests^55−59^ and some clinical trials^12,13^ have shown that a local drug delivery is safe and feasible, although characterized by some limitations in terms of final therapeutic outcomes. The failure is mainly attributed to the quick drug leakage from the tumor into surrounding healthy tissues, in relation to the complex nature of the GBM microenvironment and to the difficulties in controlling the volume of distribution of the therapeutic agent after administration.^6,7,55,64,65^ In this context, recent studies have positively proposed the use of hydrogels or polymeric nanocomposites to enhance the intratumoral drug retention and thus the treatment of orthotopic GBM tumors.^58,60^ Kang et al.^60^ demonstrated the benefits of combining the local injection of hydrogel nanocomposite containing drug-loaded micelles and ferrimagnetic iron oxide nanocubes exposed to AMF for postoperative GBM treatment. However, the spreading after the local administration and the long-term retention of multifunctional lipid-based nanocarriers in the brain cancer microenvironment have been scarcely investigated *in vivo*, and with our study we demonstrated that the proposed nanoparticles are able to reach wide areas of the tumor within a short time after administration, representing an optimal candidate for *in situ* drug delivery.

### Cytotoxic Effect of Nanovectors on GBM

**Figure 7:**
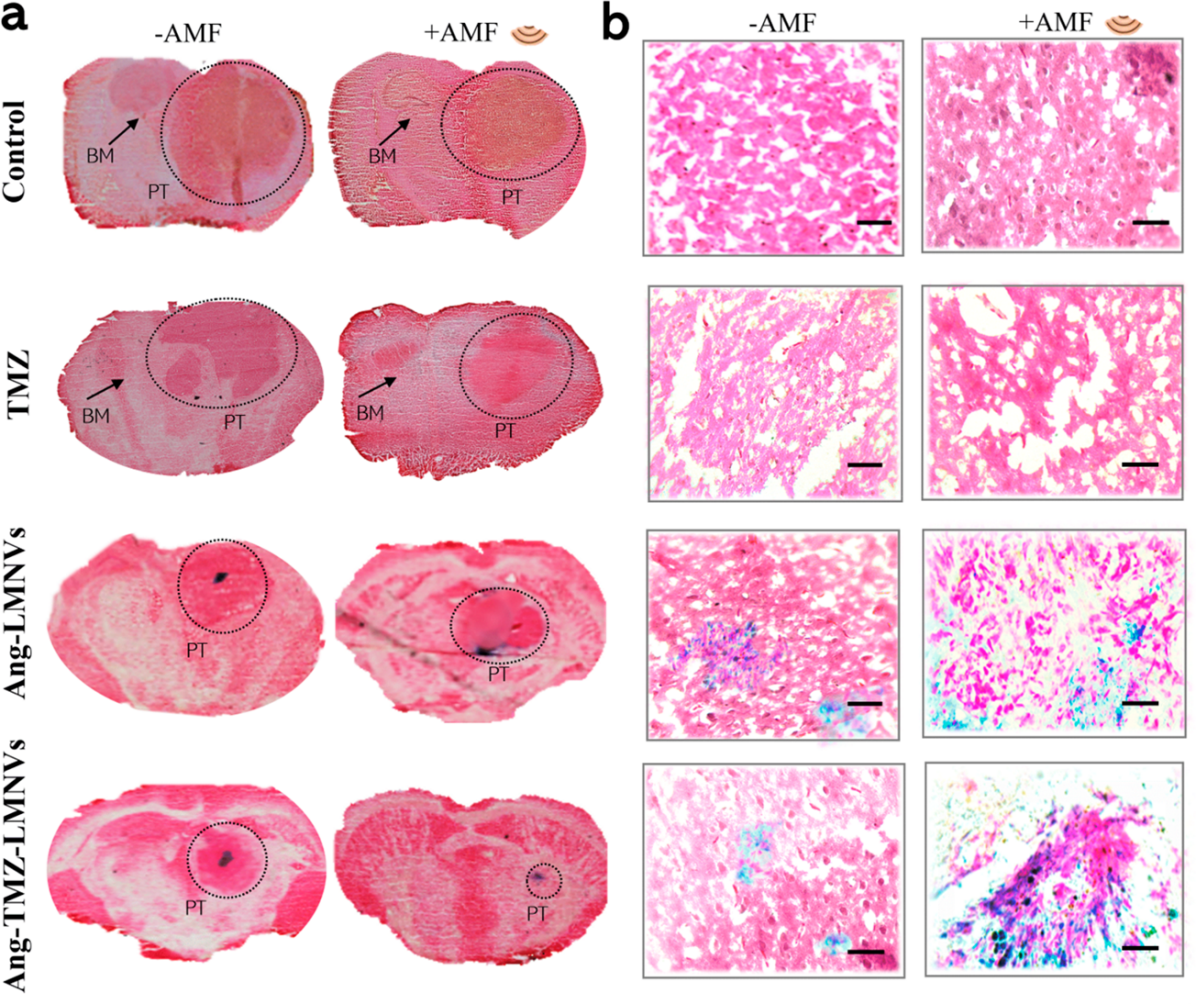
Anti-GBM effect of combined chemo-hyperthermia treatment mediated by Ang-TMZ-LMNVs + AMF. (a) Representative histological images of U-87 MG-Luc2-bearing brains excised at the end point. (b) Representative tumor sections stained with Prussian blue. Scale bar: 50 μm.

**Figure 8:**
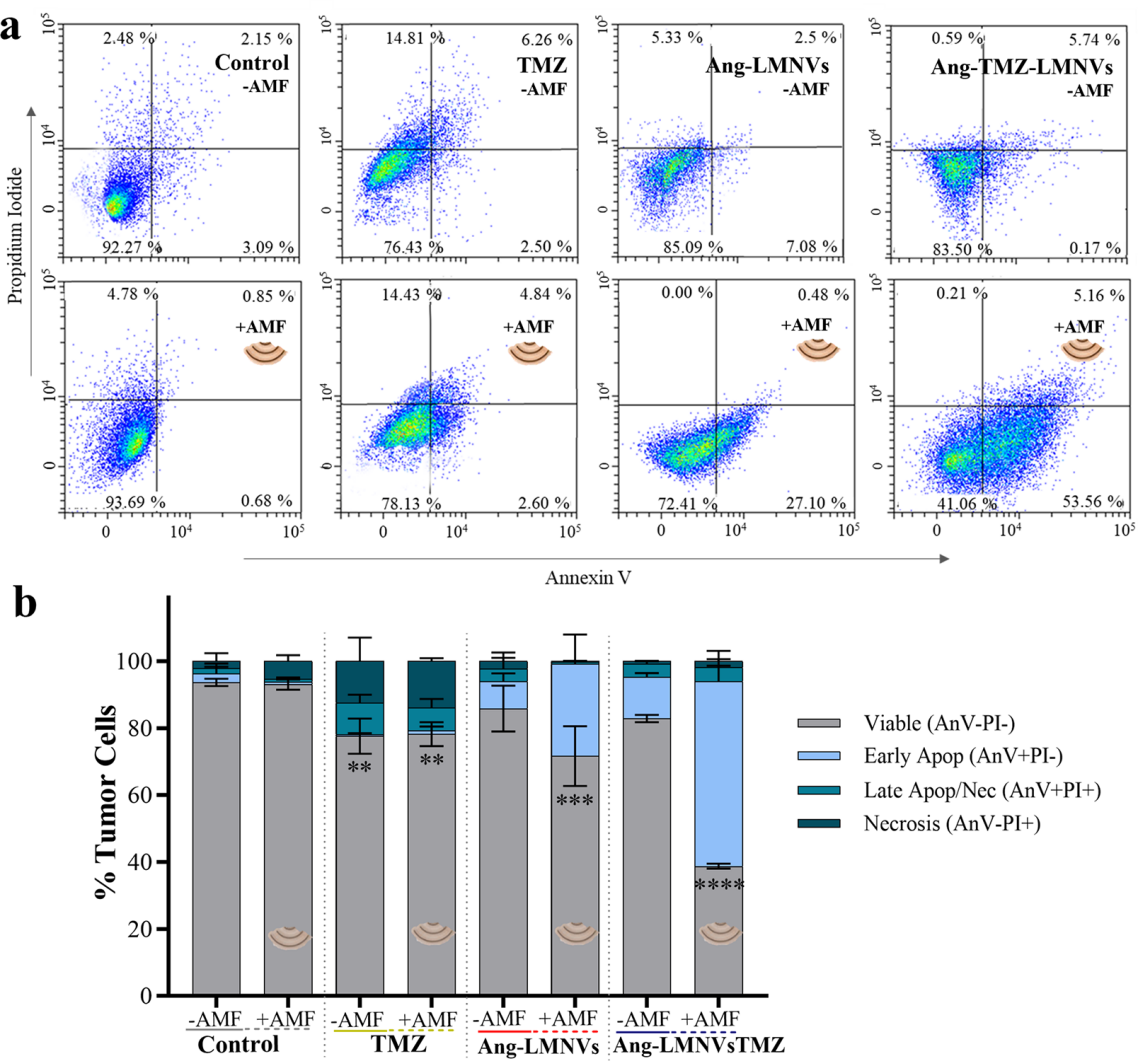
Tumor cell death (annexin V-FITC/PI staining) induced by chemo-hyperthermia treatment mediated by Ang-TMZ-LMNVs with respect to control, TMZ, and Ang-LMNVs group (with and without AMF treatment) at the experimental end point. (a) Representative scatter plots for each experimental group. (b) Quantitative analyses obtained from flow cytometry. Data are represented as mean ± SD (*n* = 3; **** p < 0.0001; ** p < 0.01; * *p* < 0.05).

Differences among experimental groups were analyzed in terms of histological features (Figure 7) and induction of cell death (Figure 8) at the experimental end point, to obtain further information on the anti-GBM effect of the proposed synergistic therapeutic approach. The location of the nanovectors in the tumor tissue and the gross morphology of the tumors were observed following a specific iron staining based on Prussian blue (Figure 7b) and on a conventional hematoxylin and eosin (H&E) staining (Figures 7a). The death rate was quantified using the commonly used test based on annexin V-FITC (AnV)/propidium iodide (PI) to determine apoptotic or nectrotic EpCam-positive cells (Figure 8).

Whole-brain reconstruction images of mice with orthotopic tumors showed that magnetic nanovectors (Ang-LMNVs and Ang-TMZ-LMNVs with or without AMF exposure) were mainly found in the central areas of the tumors at the experimental end point (blue spots indicate the presence of nanovectors, Figure 7a), consistent with our previous *in vivo* images (Figures 4 and 6). Large intracranial metastases (BM) were observed in mice (80–90% of subjects in each group) receiving saline solution (Control and Control + AMF) and free TMZ (TMZ and TMZ + AMF) (Figure 7a). Conversely, mice that received nanovectors (Ang-LMNVs and Ang-TMZ-LMNVs), even when not exposed to AMF, showed sharply delimited tumors from the brain parenchyma without metastatic signals (Figure 7a). The absence of visible brain metastases in the DLIT (Figure 6) and in the histopathological images (Figure 7a) suggests that the even plain nanovectors might have antimigratory properties, thus reducing tumor invasion, a phenomenon that could be ascribable to ferroptosis^66,67^ but that will require future dedicated investigations.

Tumor tissue histology and subsequent flow cytometry analysis further confirmed the efficient therapeutic effect of combined chemo-hyperthermia treatment induced by Ang-TMZ-LMNVs. Multiple regions of high-density cell networks surrounding a centrally pink-stained area characteristic of human GBM tissue^68^ were observed in all groups. However, a marked nuclei fragmentation (karyorrhexis) and dissolution of the nucleus (karyolysis) evidencing extensive foci of necrosis are evident in tumors treated with Ang-TMZ-LMNVs + AMF. In addition, the Ang-TMZ-LMNVs + AMF-treated group qualitatively shows enlarged intercellular spaces, not visible in the control group, and present at a lower extent in the cases of single chemotherapy and hyperthermia treatments (free TMZ and Ang-LMNVs + AFM), indicating markedly reduced tumor cell proliferation triggered by the combinatorial therapy, in agreement with the significant decrease in tumor growth observed *in vivo* (Figure 2). Surprisingly, these tissue lesions were less prounonced in the group treated with Ang-TMZ-LMNVs with respect to the treatment with free TMZ. A plausible explanation can be related to the delayed release of TMZ from the nanovectors.^69^

Eventually, the analysis of cell death by flow cytometry was consistent with histologic observations (Figure 8). The data showed a significant increase in apoptosis (≈60%) in the group treated with the combined approach (Ang-TMZ-LMNVs + AMF) compared with mice receiving saline (untreated control group, <10%) and in mice treated with free TMZ and Ang-LMNVs exposed or not to AMF (Figure 8). Noteworthy, these results confirmed that MH induced by nanovectors either as individual treatment (Ang-LMNVs + AMF) or in combination with chemotherapy (Ang-TMZ-LMNVs + AMF) mainly induced apoptosis, in contrast to chemotherapy alone, where predominantly late apoptosis/necrosis events were observed.

Besides the significant systemic side effects of conventional cancer therapies (especially chemotherapy), the limitation of their undesired effects on the central nervous system is known to be an unmet challenge in neuro-oncology.^70−73^ As an emerging alternative, several biomimetic drug delivery nanosystems capable of triggering mild MH to better control the biological consequences of the antitumor treatment have been proposed in the literature.^17,29−32,35−37^ Compared with conventional drug formulations, lipid-based nanocarriers offer significant advantages, such as better drug solubility, selective targeting, and reduced side effects.^14,15^ In turn, mild MH has been shown to effectively modulate different cell death mechanisms;^32,33^ however, it preserves the cell membrane intergrity: a phenomenon that may prevent the activation of inflammatory responses.^74^ In this scenario, our results demonstrate that Ang-TMZ-LMNVs have a long-term retention in glioma without leakage to healthy brain areas, thus presenting the potential to be used as a multifunctional nanoplatform for combined drug delivery and intratumoral MH.

Considering that almost all GBM patients will undergo maximal tumor resection as first-line therapy,^4^ local drug delivery remains an attractive tool for oncologic treatment after surgery, suitable also for patients who are not candidates for surgical resection.^10−13^ In this study, the versatility of the developed nanoplatform for future use in multiple delivery routes was addressed by functionalization with the peptide angiopep-2.^32^ This peptide is not only able to improve BBB crossing by receptor-mediated transcytosis after binding to the LRP1, but also works as a “dual targeting” agent, LRP1 being overexpressed also by glioma cells.^40,41^ The promotion of an enhanced internalization extent in cancer cells guarantees long-term residence in the tumor, avoiding spreading to healthy tissues.

This strategy would thus allow more specific and localized therapies without damaging the delicate microenvironment of the central nervous system, and, eventually, it may be potentially applicable to patients with unresectable GBM or as an alternative before or after surgery.

### Study Limitations

One of the major challenges related to combined anti-GBM nanosystems, which directly affects their preclinical outcomes, is related to their distribution pattern, often irregular and with undesirable leakage into the outer region of the tumor, even after local administration.^10−13^ As a result, treatment efficacy may be compromised due to difficulties in maintaining a therapeutically relevant dose at the target site. To tackle this issue, we tested a promising brain-penetrating nanoplatform^30−32^ that has high potential for the treatment of intracranial tumors due to its potential for glioma cell targeting and controlled release of chemotherapy. The proposed nanoplatform is designed to promote specific internalization into glioma cells, enabling remote drug release and effective tumor sensitization by heat. This provides a multimodal therapeutic strategy in a single treatment suitable to enhance specific targeting of GBM, suppress resistance by increasing tissue sensitivity to the drug, and achieve a longer residence time while ensuring minimal interaction with healthy cells.

The full exploitation of the synergy between chemotherapy and magnetic hyperthermia, combined into a single nanoplatform, is ensured by precise spatiotemporal release and effective sensitization of the tumor by heating, with a protocol optimized for *in vivo* application on an orthotopic human GBM xenograft model. To date, no preclinical studies have been performed on human tumor cells to overcome GBM resistance to chemotherapy by using this specific approach.^75,76^

Although the chosen *in vivo* model can mimic some features of human disease,^68^ one of the limitations of the present study, related to the used model, is that a more comprehensive immunohistochemical analysis could be not performed to evaluate the direct effect of the proposed treatment on markers such as Ki67 (cell proliferation) and ClC3 (cleaved caspase 3, cell death) to complement the *ex vivo* assestment. The lack of a previously established vascular network in tumor cell-derived *in vivo* models results in decreased oxygenation and limited access of nutrients, leading to the formation of an internal necrotic core in the tissue in long-term studies. This makes it difficult to obtain reliable results from immunohistochemical staining, due to a high background signal.^77,78^ Future studies could be directed toward optimizing immunohistochemistry protocols for the automated quantification of immunostaining.

Considering tumor progression monitoring, other imaging modalities such as magnetic resonance imaging (MRI), positron emission tomography (PET), or single photon emission computed tomography (SPECT), more sensitive to detect changes in brain vasculature and for drug tracking, could be used in the framework of future multicenter studies to combine the functional information here obtained with complementary structural data to fully interpret the mechanism of action of the proposed nanoplatform in the treatment of gliomas.

Eventually, we have to consider that preclinical cancer biology has largely relied on the use of human cancer cell lines and on derived xenograft tumor models to determine the therapeutic efficacy of new therapeutics.^77,78^ However, the process of establishing conventional GBM cell lines leads to irreversible loss of important biological properties of individual tumors, resulting in the failure of recapitulating the GBM heterogeneity.^79,80^ This limitation can be overcome, after preliminary proof-of-concept studies, by the establishment of *in vitro* and *in vivo* GBM models derived from patient samples, which indeed represent an intermediate step toward the clinical translation.

## Conclusions

Multifunctional biomimetic lipid nanovectors encapsulating TMZ and SPIONs, capable of inducing a potent antiglioma effect by synergistic intratumoral chemo-hyperthermia in an orthotopic human GBM mouse model, were successfully developed and tested. Our multifunctional nanoplatform did not exhibit toxicity at the tested dose, which is promising for future clinical translation. *In vivo* tracking of the nanovectors shows no presence in other organs or in the outer brain regions of the tumor, indicating that the nanovectors were effectively retained in the tumor. This is a very important aspect, because although the intratumoral chemo-hyperthermia treatment is a promising approach against GBM, drug leakage remains a major challenge that reduces the potential effect of treatment. In this regard, our study goes beyond the state of the art by approaching an effective and safe strategy that fully exploits the synergistic effects of chemotherapy and hyperthermia into a single nanoplatform with high spatial and temporal control of the treatment, and durable benefits just after a single administration. This overcomes one of the major challenges of the current clinical approaches, namely, achieving the best effects with minimal doses. The proposed biomimetic smart nanoplatform provides a multimodal “one-shot” therapeutic strategy that exhibits high cytotoxicity in human GBM by effectively sensitizing the tumor to chemo-hyperthermia treatment; our study eventually suggests the potentiality in preventing the migration of tumor cells and the formation of metastases, and in enabling specific and localized therapy with minimal to no interactions with healthy tissues.

## Experimental Section

### Synthesis and Characterization of Lipid-Based Magnetic Nanovector for In Vivo Testing

Lipid-based magnetic nanovectors (LMNVs) were synthesized through an ultrasonication/homogenization approach based on a previously reported method.^30−32^ LMNVs were cooled at 4 °C for 30 min, purified by three centrifugation steps (16 000*g*, 90 min, 4 °C), and finally redispersed in 1 mL of Milli-Q water (Millipore). Temozolomide-loaded nanovectors (TMZ-LMNVs) were obtained with an identical procedure previously described,^30,31^ by adding 2.5 mg of TMZ (Sigma-Aldrich) to the lipid mixture. Functionalized nanovectors (Ang-LMNVs and Ang-TMZ-LMNVs) were obtained by conjugating angiopep-2 (Selleckchem) following an optimized protocol.^32^ At the end of the conjugation reaction, functionalized nanovectors were washed by centrifugation (16 000*g*, 90 min, 4 °C), and the final pellet was redispersed in 1 mL of Milli-Q water. Ang-LMNVs and Ang-TMZ-LMNVs were labeled with the near-infrared (NIR) fluorescent DiOC_18_(7) dye (DiR, Invitrogen) by incubating 1 mg of particles with 5 μL of dye (1 mg/mL in dimethyl sulfoxide (DMSO)) for 2 h at 37 °C and then washing three times by centrifugation (16 000*g*, 90 min, 4 °C).

LMNVs loaded or not with TMZ were already characterized in previous studies^30−32^ in terms of morphology (transmission electron microscopy (TEM), JEOL Jem-1011; high-angle annular dark field-scanning transmission electron microscopy (HAADF-STEM), TEM JEOL JEM-2200FS), of stability at different temperatures, and in aqueous media with different conductivities and ionic strengths (dynamic light scattering (DLS), Zetasizer NanoZS90 Malvern Instruments) as well as in terms of physical (thermogravimetric analysis (TGA); Q500 analyzer) and magnetic properties (superconducting quantum interference device (SQUID), Quantum Design). Drug release features (at different physiological conditions and with/without the application of an AMF) have been evaluated through high-performance liquid chromatography (HPLC, Shimadzu LC-20AT), and, eventually, long-term stability in complete culture medium was evaluated by dynamic light scattering (DLS, Zetasizer NanoZS90 Malvern Instruments).

Hyperthermia measurements were carried out using a MagneTherm (NanoTherics) equipped with a round coil of 9 turns/44 mm inner diameter and B11 capacitor. Samples were placed inside a 2 mL Eppendorf tube, inserted in a polystyrene case in order to reduce thermal fluctuations, and then exposed to AMF (10–16 kA/m; 334–750 kHz) for 30 min. The temperature of the sample was measured using an infrared camera (Fluke Ti200 IR-Fusion technology) placed perpendicularly to the surface to avoid visual angle error. Before the application of the AMF, the temperature was measured for 60 s to ensure thermal stability. The distance between the IR camera and sample was maintained at 0.1 m for each acquisition.

The effect of the nanovectors on red blood cell (RBC) integrity was evaluated following a standard assay previously reported.^81^ Briefly, preserved blood collected from euthanized mice (0.9–1 mL per mice, *n* = 3; authorization 746/2021-PR of the Italian Ministry of Health) was processed by adding 3.8% sodium citrate solution followed by centrifugation at 1000*g* for 10 min, until obtainment of a clean RBC pellet, washed 3 times with 0.9% NaCl. After the last wash, RBCs were gently resuspended in saline solution to obtain a 5% RBC suspension. Then, 400 μg/mL of either Ang-TMZ-LMNVs or Ang-LMNVs was incubated at 37 °C with the RBC suspension by gently shaking on an orbital plate shaker. A positive control consisting of 50% v/v deionized water and a negative control consisting of a 5% v/v phosphate-buffered saline (PBS) solution in RBC suspension were also analyzed. After incubation, tubes were centrifuged at 500*g* for 5 min. The absorbance of hemoglobin released in the supernatants was determined at 24 and 72 h using a microplate reader (Tecan Infinite M200) at 540 nm. Hemolysis percentage was calculated by normalizing all experimental results to the mean absorbance value of the positive control (100% hemolysis).

### Cell Line and Cell Culture

Luciferase-expressing human glioblastoma (GBM) cell line U-87 MG-Luc2 (HTB-14-LUC2 ATCC) was cultured and maintained at 37 °C in a humidified atmosphere with 5% CO_2_ using Dulbecco’s modified Eagle’s medium (DMEM, ThermoFisher Scientific) supplemented with 10% fetal bovine serum (Gibco FBS, ThermoFisher Scientific), 1% antibiotic–antimycotic (Gibco 100×, ThermoFisher Scientific), and l-glutamine (200 mM, ThermoFisher Scientific), named as “complete DMEM” (cDMEM). To detach the cells, a two-step protocol was followed to try to enhance the cell viability. First, cells were incubated with a 0.05% trypsin/ethylenediaminetetraacetic acid (EDTA) solution for 1 min at 37 °C; then, the cells were gently scraped from the culture plate. Finally, cells were collected in fresh cDMEM.

### Orthotopic Xenograft Tumor Model

Mice (*Crl*:*NU-Foxn1*^*nu*^) were commercially obtained from Charles River Laboratory and were maintained in the Animal Facility (AF) of *Fondazione Istituto Italiano di Tecnologia*, IIT. Before the start of each procedure, mice were held for acclimation during 1 week after arrival to the AF. Mice were housed in individually ventilated cages (IVCs) in a temperature-controlled room with a 12/12-h dark-light cycle and had *ad libitum* access to water and low fluorescence diet (4RF21, Mucedola). Animals were housed in accordance with IIT institutional regulations for animal care, welfare, and health care. The health and welfare of the animals were regularly checked by a veterinarian. All efforts were made to minimize animal suffering and to use the minimal number of animals required to produce reliable results according to the “3Rs concept”. All animal experiments were performed in compliance with EU Directive 63/2010 and Italian Law 26/2014 and approved by the Ethics Committee (authorization 746/2021-PR of the Italian Ministry of Health), in accordance with the recognized national regulations for the protection of animals used for scientific purposes.

To establish an orthotopic brain tumor model, 5–6 weeks old pathogen-free female immunodeficient nude mice were first anesthetized by inhalation of isoflurane (4% for the induction and 2% for the maintenance). Then, a burr hole was drilled in the skull 1.5 mm posterior to the bregma and 1.4 mm lateral to the midline. Afterward, U-87 MG-Luc2 cells (1 × 10^5^ cells/3.0 μL cDMEM) were intracranially injected using a stereotaxic instrument equipped with a 10 μL Hamilton syringe with a 27-gauge needle. Tumor cells were slowly injected into the brain tissue at a depth of 3.0 mm from the brain surface using a microinjection autopump (rate 0.25 μL/min). At the end of the procedure, the needle was gently withdrawn, and the surgical incision was closed with adhesive glue for tissue (3M Vetbond Tissue Adhesive, 3 mL, ThermoFisher Scientific). A warming pad was used during the entire procedure to minimize animal heat loss, and a sterile 0.9% NaCl physiological saline solution was subcutaneously injected to avoid possible dehydration. Analgesics and antibiotics were also administered during the postoperative and peri-operative periods, under the strict supervision of the veterinary surgeon, to minimize any possible distress and suffering. A warming lamp was used to promote postoperative recovery.

### In Vivo Treatments

Mice bearing U-87 MG-Luc2 human cell orthotopic xenografts were maintained under weekly observation until the tumor signal reached the plateau phase. Then, they were randomly divided into eight groups (*n* = 8/group) treated by a single intratumoral injection with physiological saline solution (Control), free temozolomide (0.98 mg/kg_weight_), Ang-LMNVs (24 mg/kg_weight_), and Ang-TMZ-LMNVs (24 mg/kg_weight_, corresponding to [TMZ] = 0.98 mg/kg_weight_), either treated or not with an AMF. In all cases the final injection volume was 3 μL, and the same surgical procedure previously described for the tumor induction was followed. Twenty-four hours after injection, mice belonging to the MH treatment groups were exposed to an AMF (*f* = 334.1 kHz; *H* = 12.67 kA/m; *t* = 30 min) for 3 consecutive days by using a commercial AMF generator (MagneTherm system equipped with a round coil of 17 turns/44 mm inner diameter and B22 capacitor) housed inside the Animal Facilities. Mice were anesthetized with intraperitoneal ketamine/xylazine (100 mg/kg_weight_/10 mg/kg_weight_) during the experimental procedure. After each treatment, mice were transferred to the original cage, and a warming lamp was used to promote their recovery until awakening. After the last AMF exposure, mice were maintained to evaluate their response to the treatment. The weight of the mice was followed 3–4 times weekly during all experimental courses as the main indicator of their wellness.

### Assessment of Therapeutic Efficacy and LMNV Location by In Vivo Imaging

The tumor evolution and the nanovector location were followed through bioluminescence and fluorescence imaging, using the noninvasive *In Vivo* Imaging System (IVIS Spectrum, PerkinElmer) under anesthesia with oxygen-isoflurane (4% for the induction and 1% for the maintenance). Luciferase-labeled U-87 MG-Luc2 tumor cells allowed us to follow the tumor evolution over time. To obtain the bioluminescence images, awake mice were *i.p.* injected (150 mg/kg_animal_) with D-luciferin (15 mg/mL), and after 10–12 min, the acquisition was performed by using the Living Image 4.5.1 software. Fluorescence images of Ang-LMNVs and Ang-TMZ-LMNVs were acquired with the DiR filter (λ_ex_ = 750 nm, λ_em_ = 782 nm) of the same equipment. 3D diffuse tomography (DLIT) and 3D fluorescent imaging tomography (FLIT) modalities of IVIS were also used to image the whole-body mice and the brain area. The grayscale photographs and bioluminescent/fluorescence images of the mice were overlaid and analyzed using the Living Image 4.5.1 software. Regions of interest (ROI) were drawn over the signals, and average (Avg) radiant efficiency was quantified by the sum of the radiance (photons) from each pixel inside the ROI/number of pixels or super pixels (p/s/cm^2^/sr). Efficiency of fluorescent emission (epifluorescence) was normalized to the incident excitation intensity and expressed as p/s/cm^2^/sr/μW/cm^2^. DLIT and FLIT data sets were subjected to 3D tomographic reconstruction to generate axial, coronal, and sagittal bioluminescence and fluorescence images. In addition, the 3D DLIT images were analyzed using the integrated 3D software tool to calculate the total flux (photons/s), source volume (mm^3^), and source depth (mm) according to the manufacturer’s instructions. After image acquisitions, animals were allowed to recover from anesthesia in their cage.

### Histological Procedures

Anesthetized mice were euthanized by cervical dislocation when critical disease symptoms were reached^62^ or at the end point of the experiment (70 days after treatment initiation), and the entire brains were collected. Frozen brain sections embedded in FSC22 (Surgipath, Leica Microsystems) and cut into serial 10 μm sections (Leica Cryostat CM1860UV) were fixed with a 4% paraformaldehyde solution (Fisher, SC 281692) for 20 min and washed with PBS before histochemical staining. Nanovectors were localized by staining with potassium ferrocyanide (Perl’s Prussian Blue) using the Iron Stain Kit (Abcam, ab150674) according to the manufacturer’s protocol. Adjacent sections were stained with hematoxylin (H) and eosin (E), according to the standard protocol for cryosectioned tissue. Nuclei were stained by immersion in a hematoxylin solution (GHS232, Sigma) for approximately 3 min. Then, the excess was washed with distilled water (2×, 1 min), followed by successive immersions in acid ethanol (one rapid immersion) and ammonia–water (10–20 immersions, 1 min); each immersion was alternated with washes in distilled water (2×, 1 min). The cytoplasm was stained by immersion in eosin (HT110132, Sigma) for 45 s (2–10 immersions). Brain sections were then washed with distilled water (3×, 1 min) and dehydrated by successive immersions in 70% ethanol (2 min), 80% ethanol (2 min), 90% ethanol (2 min), and then in 100% ethanol (ECSA Chemicals, RE 30860; 2×, 1 min). Finally, brain sections were immersed twice in fresh xylene (534056, Sigma-Aldrich) for 1 min to complete fixation and water exclusion. Stained tissue sections were mounted with SP15100 Permount Mounting Medium (Fisher Chemical) and imaged by using a Leica DM5500 B microscope (software LEICA LAS-X, camera DMC 2900 Color, objectives HCX-PL-FLUOTAR 5×/0.15, HC-PL-FLUOTAR 10×/0.3, HC-PL-APO–CS 20×/0.7; Leica Microsystems). Image reconstructions were performed using the Neurolucida system with a 4×/0.13 objective (UPLFLN4XPHP, Olympus).

### Extraction and Isolation of Single Cell from Orthotopic Brain Tumors

The excised brain tumors were kept on ice in DMEM. They were then placed in a 6 cm plate containing 3 mL of Liberase (TM Research grade, Sigma-Aldrich), cut into small pieces with a scalpel, transferred to a 15 mL Corning tube, and incubated for 15 min at 37 °C under constant shaking. After the reaction was stopped (with the addition of 1 mL of FBS/9 mL of DMEM), the suspension was centrifuged at 300*g* for 5 min. The resulting pellet was gently mixed with 2 mL of trypsin/EDTA and placed in a shaker incubator at 37 °C for 3 min. The reaction was neutralized by adding 2 mL of FBS/13 mL of DMEM and then centrifuged (5 min at 300*g*), discarding the supernatant. The pellet was resuspended in 5 mL of DMEM before being filtered through a 70 μm cell strainer into a 50 mL tube. The remaining large pieces of tissue were processed through the cell strainer with the wide end of a 1 mL plastic syringe. The single cell suspension was transferred into a 15 mL tube before centrifugation at 300*g* for 5 min. The resulting pellet was resuspended in 0.8 mL of 1× RBC Lysis Buffer (ab204733, Abcam) by continuous shaking with a 1000 μL pipet for 20–30 s. The reaction was stopped by adding DMEM (5–10 mL), the cells were washed (5 min at 300*g*), and the obtained pellet was resuspended in the desired volume of appropriate buffer for later analysis.

### Flow Cytometry Analysis

To examine the percentage of selected tumor markers, single cells were resuspended in a blocking solution (ice-cold PBS containing 10% FBS and 1% sodium azide) at a concentration of 1 × 10^6^ cells/mL. They were then incubated at 4 °C for 30 min in the dark with 0.1 μg/mL α-EpCAM-PE CD36 antibody (ab112068, Abcam; λ_ex_ = 488 nm, λ_em_ = 575) and isotype-matched control PE mouse IgG1 [B11/6] (ab91357, Abcam; λ_ex_ = 488 nm, λ_em_ = 575). Cells were then washed three times by centrifugation (300*g*, 5 min) and resuspended in 0.5 mL of blocking solution.

Cell death induced by the different treatments was determined using an Apoptosis Kit (Thermo Fisher) based on annexin V-FITC and propidium iodide (PI). Cells were resuspended in the annexin V-FITC-binding buffer supplied with the kit containing 1 μg/mL of PI and annexin V-FITC 7 mM for 15 min (0.1 mL total volume). At the end of the incubation, 0.4 mL of the binding buffer was added to each sample, and the fluorescence of the cells was measured (annexin V-FITC: λ_ex_ = 488 nm, λ_em_ = 525 ± 40 nm; PI: λ_ex_ = 488 nm, λ_em_ = 610 ± 20 nm).

The nanovector accumulation in the tumor cells was analyzed using the PCy7 channel (λ_ex_ = 488 nm, λ_em_ = 780 ± 60 nm). All flow cytometry analyses were performed using a Cytoflex (Beckmann Coulter), and data were interpreted by using the CytExpert 2.5 software (Beckman Coulter).

### Statistical Analysis

Data collected were expressed as mean ± SD. Statistical significance was determined by one-way analysis of variance (ANOVA) using GraphPad Prism v7.00 software. The confidence interval was 95%. Sidak’s and Dunnet’s multiple comparison tests were used, and significant values were expressed as ****p < 0.0001, **p < 0.01; *p < 0.05, *p* > 0.05 no significance.

Median survival times (*T*_MS_) of mice receiving different treatments were analyzed using the Kaplan–Meier survival curves, and statistics analysis for the overall condition was performed by using the log-rank test (Mantel-Cox).

## Supporting Information Available

TEM images and long-term stability evaluation of the nanovectors; in vitro hemocompatibility; heating capacity assessment; tumor growth assessment after orthotopic U-87 MG-Luc2 cells implantation; flow cytometry analysis of nanovector uptake efficiency in brain tumor cells; long-term stability data (PDF)
