# Supplementary material for: Drug-Loaded Lipid Magnetic Nanoparticles for Combined Local Hyperthermia and Chemotherapy against Glioblastoma Multiforme
Source: ACS Nano. 2023 Sep 12;17(18):18441–55. doi: 10.1021/acsnano.3c06085 (PMC10540267; doi:10.1021/acsnano.3c06085)
Supplement: Supplementary file 1 — nn3c06085_si_001.pdf [file nn3c06085_si_001.pdf]

SUPPORTING INFORMATION

Drug-Loaded Lipid Magnetic Nanoparticles for Combined Local Hyperthermia and Chemotherapy against Glioblastoma Multiforme

Lilianne Beola[*±], *Nerea Iturrioz-Rodríguez*[±,†], *Carlotta Pucci*[±], *Rosalia Bertorelli*[#], *Gianni Ciofani*[*±]

[±]Smart Bio-Interfaces, Istituto Italiano di Tecnologia, Viale Rinaldo Piaggio 34, Pontedera, 56025 Italy
[#]Translational Pharmacology, Istituto Italiano di Tecnologia, Via Morego 30, Genova, 16163 Italy

[†]*Present Address:* Cellular Oncology Group, Biodonostia Health Research Institute, P° Dr. Beguiristain s/n, Donostia-San Sebastian, 20014 Spain

*Corresponding Authors:
e-mail: lilianne.beolaguibert@iit.it; gianni.ciofani@iit.it

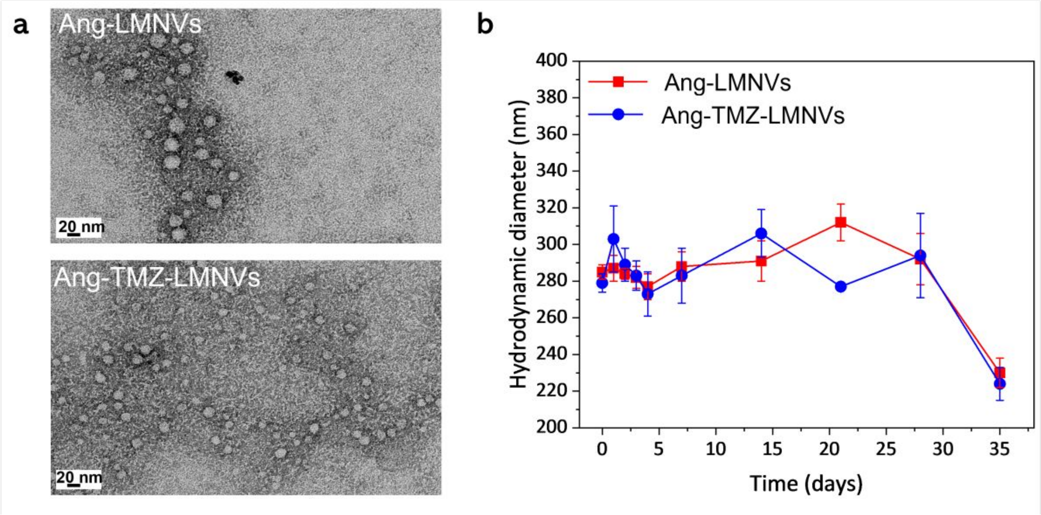

Figure S1. Physicochemical characterization of lipid magnetic nanovectors. (A) Representative TEM images of Ang-LMNVs and Ang-TMZ-LMNVs. (B) Stability over time in terms of hydrodynamic diameter (nm) for Ang-LMNVs (red squares) and Ang-TMZ-LMNVs (blue circles) incubated with DMEM + FBS (10%) at 37°C.

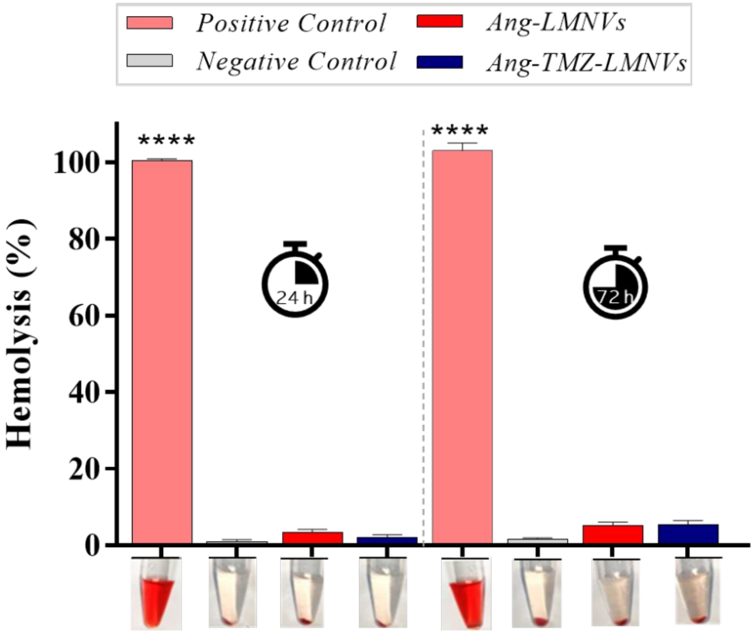

Figure S2. *In vitro* hemocompatibility testing of lipid magnetic nanovectors. Assessment of the percentage of hemolysis induced by Ang-LMNVs or Ang-TMZ-LMNVs at different time points. Data are presented as mean ± SD ($n = 3$). Representative photographs of the results are included below the graph. Red color in the supernatant of the vials indicates damaged erythrocytes (release of hemoglobin). Purified erythrocytes were incubated with 50% v/v deionized water (positive control), 5% v/v saline solution (negative control), 400 µg/ml Ang-LMNVs, or 400 µg/ml Ang-TMZ-LMNVs (**** $p < 0.0001$ with respect to the negative control).

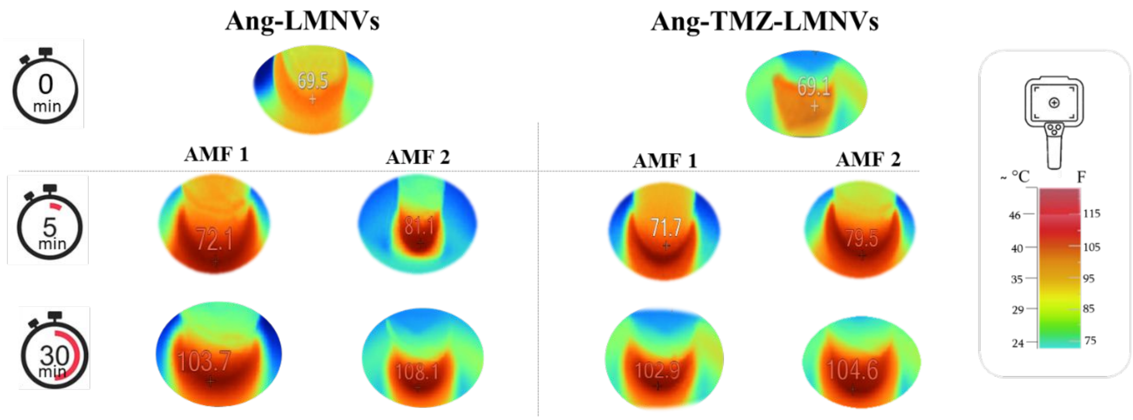

Figure S3. Evaluation of the heating capacity of lipid magnetic nanovectors after 5 and 30 min under different AMF conditions (AMF1: f = 468.2 kHz and H = 9.72 kA/m; AMF2: f = 334.1 kHz and H = 12.67 kA/m). The images are representative of two independent measurements.

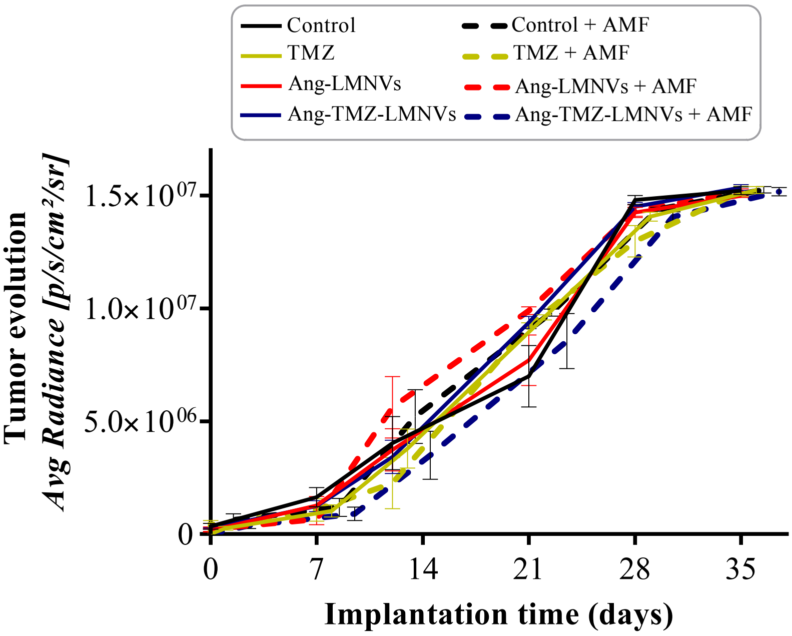

Figure S4. Tumor growth after orthotopic cell implantation, monitored through luminescence assessed with IVIS.

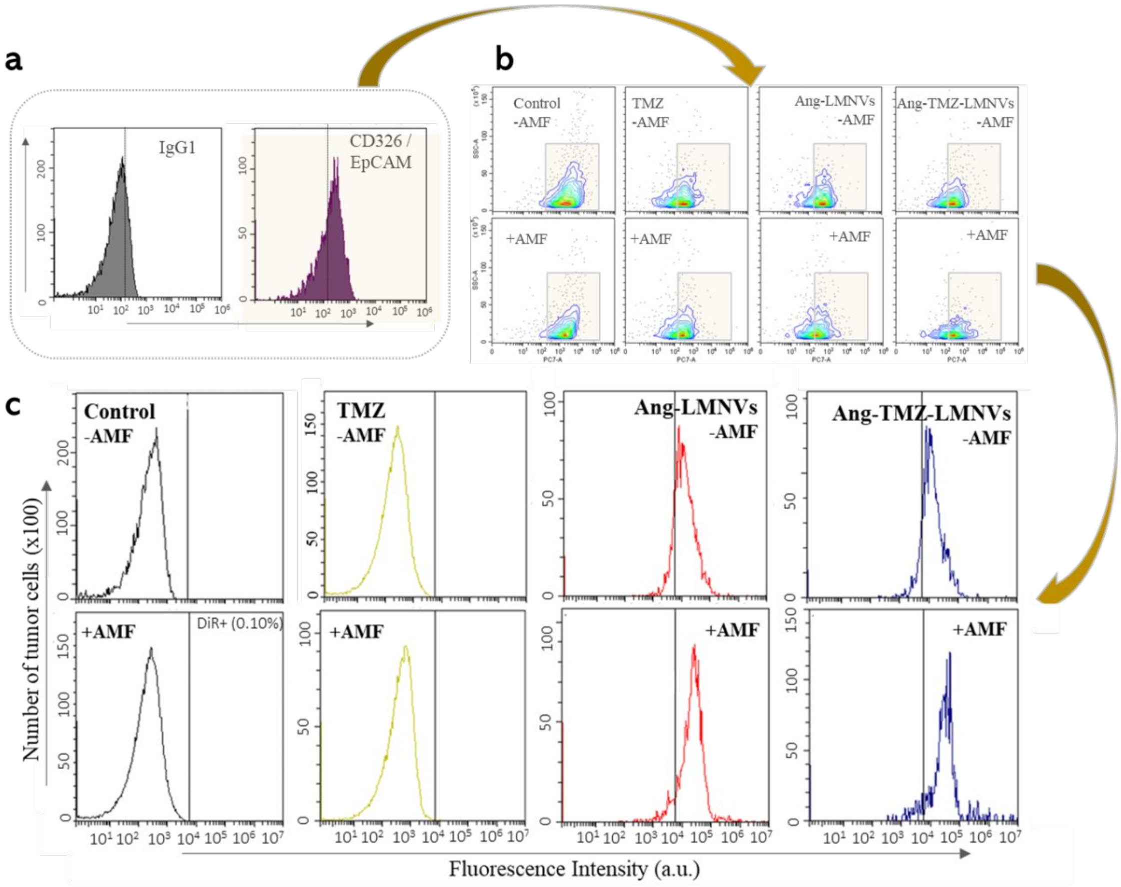

Figure S5. Flow cytometry analysis of uptake efficiency of Ang-LMNVs and Ang-TMZ-LMNVs labeled with DiR in EpCAM-positive brain tumor cells with respect to control and TMZ group (with and without AMF treatment). a) Isotype control; b) Representative contour plot of the cells selected for uptake efficiency analysis; c) Representative histograms of the DiR fluorescence signal.

	**Ang-LMNVs**		**Ang-TMZ-LMNVs**	
	**Rd (nm)**	**PdI**	**Rd (nm)**	**PdI**
**Day 0**	285 ± 4	0.302 ± 0.017	279 ± 5	0.246 ± 0.009
**Day 35**	230 ± 8	0.399 ± 0.023	224 ± 5	0.380 ± 0.008

Table S1. Summary of hydrodynamic diameter (Rd) and polydispersity index (PdI) values derived from the DLS cumulant analysis.
